# Short- and long-term impacts of variable hypoxia exposures on kelp forest sea urchins

**DOI:** 10.1038/s41598-020-59483-5

**Published:** 2020-02-14

**Authors:** Natalie H. N. Low, Fiorenza Micheli

**Affiliations:** 1 Hopkins Marine Station, Stanford University, Pacific Grove, CA 93950 USA; 2 Stanford Center for Ocean Solutions, Pacific Grove, CA 93950 USA

**Keywords:** Climate-change ecology, Ecophysiology

## Abstract

Climate change is altering the intensity and variability of environmental stress that organisms and ecosystems experience, but effects of changing stress regimes are not well understood. We examined impacts of constant and variable sublethal hypoxia exposures on multiple biological processes in the sea urchin *Strongylocentrotus purpuratus*, a key grazer in California Current kelp forests, which experience high variability in physical conditions. We quantified metabolic rates, grazing, growth, calcification, spine regeneration, and gonad production under constant, 3-hour variable, and 6-hour variable exposures to sublethal hypoxia, and compared responses for each hypoxia regime to normoxic conditions. Sea urchins in constant hypoxia maintained baseline metabolic rates, but had lower grazing, gonad development, and calcification rates than those in ambient conditions. The sublethal impacts of variable hypoxia differed among biological processes. Spine regrowth was reduced under all hypoxia treatments, calcification rates under variable hypoxia were intermediate between normoxia and constant hypoxia, and gonad production correlated negatively with continuous time under hypoxia. Therefore, exposure variability can differentially modulate the impacts of sublethal hypoxia, and may impact sea urchin populations and ecosystems via reduced feeding and reproduction. Addressing realistic, multifaceted stressor exposures and multiple biological responses is crucial for understanding climate change impacts on species and ecosystems.

## Introduction

Global climate change is altering both the intensity and patterns of variability of environmental stress that marine species, communities, and ecosystems experience. For example, increasing mean temperatures occur alongside longer, more frequent, and more extreme heat waves^1^, and long-term ocean acidification is occurring together with changes in seasonal pH variation^2^. Advances in remote sensing and *in situ* sensors are revealing tremendous variability in the physical environment that marine organisms experience, but it is challenging to disentangle the effects of different drivers and variability features in the field, and controlled laboratory experiments addressing climate change effects have primarily focused on mean ocean conditions^3^. It is important to understand how multi-faceted and dynamic aspects of environmental change will impact species and ecosystems in order to inform scientific predictions, management, and policy decisions.

Changes in the mean and extreme values of environmental conditions can increase the intensity of physiological stress that marine organisms face^4^. This can induce direct mortality when lethal physiological thresholds are crossed^5,6^. However, less-extreme, sublethal thresholds are more likely to be crossed^7,8^, causing changes in organisms’ metabolic processes, behaviour, and movement, and potentially imposing ‘cost-of-living’ consequences for key processes like growth and reproduction and the trade-offs between them^9,10^, which can lead to longer-term population impacts. Such sublethal impacts can also extend to the ecosystem level. Changes to organisms’ consumption rates, behavior, or movement patterns can scale up to impacts on ecosystem structure and dynamics if the organism plays a key functional role in its ecosystem. For example, changes in water temperature alter keystone predation rates in the intertidal seastar *Pisaster ochraceus*, potentially altering the seastar’s keystone role in maintaining benthic diversity in the rocky intertidal zone^11,12^. Acidification at natural CO_2_ underwater vents affects the movement and grazing of sea urchins, with cascading effects on benthic communities of temperate rocky reefs^13^.

Patterns of variability in environmental stress can significantly mediate lethal and sublethal impacts on organisms. For example, natural high-frequency patterns of temperature and pH variability can increase performance and physiological tolerances of some marine organisms^14–16^. Conversely, some regimes of fluctuating exposures to low pH can also be more energetically stressful than constant exposures to the same conditions^17^. Global change is altering the natural variability in environmental conditions across ecosystems^2^, and local-scale processes can further mediate these changes to produce small-scale variation in the temporal patterns of organisms’ exposure to physiological stress, potentially creating local stressor hotspots or refuges^2,18–22^. Therefore, it is important to understand how different patterns of variability in climatic stressors impact organisms and their roles in ecosystems. However, most studies on variability have focused on short-term lethal and sublethal impacts of a few key stressors (particularly warming and acidification) under constant exposure, and do not address the effects of different patterns of variability (but see^15,17,23^). A better understanding of the impacts of realistic patterns of temporally variable exposure to environmental stress is urgently needed. While ideally such understanding will eventually extend to realistic field settings, controlled laboratory experiments are a first robust approach to starting to address the complexity of variable stressor exposure.

Here we explore the consequences of different exposure patterns to sublethal physiological stress for a novel climate stressor, upwelling-driven hypoxia, on the diverse, productive kelp forest ecosystems of the California Current System by investigating, under controlled laboratory conditions, the short- and long-term responses of a key kelp grazer in these ecosystems. Globally, ocean deoxygenation is increasing with climate change^24^. In the California Current system, upwelling-driven coastal hypoxia has been recorded on the continental shelf since the early 2000s^6,25,26^, and it is expected to increase in severity as climate change intensifies upwelling processes^27,28^ and ocean deoxygenation^29^. Lethal exposures have led to a few mass mortalities in some nearshore California Current ecosystems^6,25,30^, but sublethal exposures and impacts are likely to be much more common^8^. Although large-scale upwelling processes are responsible for advecting deep, offshore hypoxic water towards the surface, smaller-scale nearshore physical and biological processes interact with local bathymetry to generate highly variable patterns of exposure to hypoxia in coastal habitats^19,26,31^, which can differ significantly even at small spatial scales^19,21,22^. However, the consequences of these sublethal and temporally variable hypoxia exposures for nearshore California Current species and ecosystems are not well known.

We focus on the purple sea urchin (*Strongylocentrotus purpuratus*) as an abundant and functionally important member of California Current kelp forest communities. Understanding hypoxia impacts on these sea urchins can shed light on how kelp forest ecosystems may be affected. Sea urchin grazing is a major contributor to kelp loss, and overpopulation and overgrazing by this species has led to kelp deforestation and ecosystem state shifts to urchin barrens in multiple California Current kelp forests^32–34^. *S. purpuratus* has seen recent population increases in multiple kelp forests across the California Current System, with reports of spatially patchy kelp deforestations^35^. Purple sea urchins also serve as prey for a diverse group of predators such as sea otters, predatory fish, and crustaceans, and can act as an important link to transfer energy up the food web from primary producers to predators^36^. Therefore, understanding the impacts of variable patterns of sublethal hypoxia on sea urchins will not only provide information on how these organisms may respond to sublethal and fluctuating climate stressors, but it can also shed light on how kelp forest ecosystems may be affected.

Purple sea urchins are tolerant of severe hypoxia and are unlikely to experience direct mortality under current and near-future conditions, but experience sublethal impacts on grazing rates at dissolved oxygen concentrations that are relatively common in California Current kelp forests^8^. Here, we examined the impacts of different exposure patterns to sublethal hypoxia on *S. purpuratus*, based on observed variability in oxygen concentrations in the field. Specifically, we quantified and compared short-term (3-day) and longer-term (9-week) responses of purple sea urchins to a constant and two fluctuating sublethal hypoxia regimes to ask: (1) What are the effects of sublethal hypoxia on sea urchin individuals, populations, and their ecosystems? (2) What are the effects of constant versus fluctuating exposure regimes? and (3) What are the effects of different fluctuating exposure regimes?

## Methods

### Collection and maintenance of organisms

We collected purple sea urchins, *Strongylocentrotus purpuratus*, from kelp forest habitat in southern Monterey Bay adjacent to the Hopkins Marine Station (36.6215°N, 121.9015°W). Sea urchins were transported to flow-through aquaria at the Hopkins Marine Station and held in flowing seawater with ambient temperature, dissolved oxygen, and pH (13.9 ± 1.1 °C, 8.39 ± 0.74 mg/L, 7.88 ± 0.08 pH) and a 12 h:12 h light:dark regime. They were maintained on an *ad libitum* diet of giant kelp (*Macrocystis pyrifera*) and allowed to acclimate for at least 4 weeks before experiments.

### Dissolved oxygen treatments

**Figure 1:**
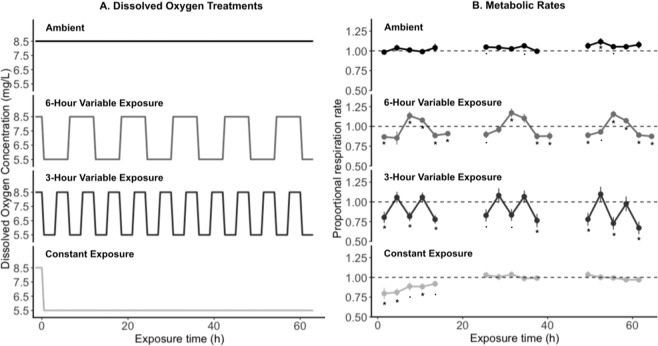
(**A**) Dissolved oxygen concentrations (mg/L) over time in each treatment. (**B**) Sea urchin metabolic rates as a proportion of their baseline (±SE). The dashed line indicates baseline (ambient) metabolic rates. Dots (.) and asterisks (*) denote values that were different from the baseline at the α = 0.1 and α = 0.05 significance levels respectively, based on one-sample t-tests.

To assess the effects of different patterns of sublethal hypoxia exposure on sea urchins, we designed four dissolved oxygen (DO) treatments (Fig. 1A): constant ambient DO conditions (“Ambient”; 8.5 mg/L), constant exposure to low DO (“Constant Exposure”; 5.5 mg/L), and two treatments in which DO fluctuated between ambient (8.5 mg/L) and low (5.5 mg/L) levels for periods of 3 or 6 hours (“3-Hour Variable” and “6-Hour Variable” respectively). The total duration of low DO exposure for these treatments was exactly half of that in the constant exposure treatment. We chose 5.5 mg/L as our low DO level because purple sea urchins are known to experience sublethal impacts at this threshold^8^. Both variable exposure treatments were chosen to approximate aspects of field DO variability based on a decade-long DO dataset from nearshore Monterey Bay^8,26^. The mean duration of exposure to 5.5 mg/L of DO was about 3.3 hours^8^, and spectral analysis of the dataset showed a dominant semidiurnal signal in DO variability^26^. Continuous exposures to this sublethal threshold have also occurred for up to 4 weeks, although they are uncommon (Low *et al*., unpublished data). Therefore, we chose exposure cycles of 3 and 6 hours for our variable exposure treatments. Having two different variable exposure treatments with the same total exposure duration also allowed us to directly examine whether the pattern of variability, not just total exposure duration, could modulate impacts.

We used these four DO treatments in three experiments to assess short- and long-term impacts of exposure to low DO: (1) a 3-day metabolic rate experiment; (2) a 3-day grazing experiment; and (3) a 9-week experiment to measure growth, calcification, and gonad production. We manipulated DO within 189-L insulated aquarium tanks supplied by flow-through seawater. These served as header tanks for respirometry chambers in the metabolic rate experiment, and as experimental aquaria in the other two experiments. To establish and maintain DO treatments, we bubbled nitrogen gas into aquaria seawater through 60 mm fine-pore air diffusers. The flow of nitrogen gas was controlled by solenoid valves connected to an Arduino microcontroller system (Low *et al. in review*), which continuously monitored seawater DO concentrations using an optical DO probe (Vernier Software and Technology) and initiated feedback loops to maintain DO concentrations at treatment levels. DO levels in the tanks were verified every 1–2 days using a handheld DO probe (YSI Pro Plus, Xylem Inc.).

### 3-day metabolic rates

We measured oxygen consumption rates of *S. purpuratus* in each DO treatment using intermittent-flow respirometry from October 2016 and February 2017. Sea urchins (test diameter 25–35 mm) were fasted for 5 days before the start of each trial, and then placed individually in 0.47-L respirometry chambers containing an optical DO probe (Vernier Software and Technology). The chambers were kept in a 14 °C water bath, supplied with treatment seawater from a header tank, and well-mixed using magnetic stirrers.

We ran two trials with six sea urchins each per DO treatment, for a total of 12 replicates per treatment. In each trial, we measured oxygen consumption rates for each sea urchin 1.5 hours before the start of each treatment to establish individual baselines, and then at 3-hour intervals for 4–5 time points per day during the 3-day treatment (see Fig. 1B). We recorded one additional time point per day for the 6-hour variable treatment in order to sample across a full period of DO variation. At each time point, we halted the flow of seawater to the respiration chambers, and measured the change in DO concentrations in the closed chambers as sea urchins depleted the oxygen. When approximately 10% of DO was consumed, we flushed the chambers with treatment seawater and repeated the recording process to give two replicate recordings of oxygen consumption for each sea urchin at each time point.

After each trial, sea urchins were removed from chambers and euthanized, dried at 60 °C to constant mass for 36–48 hours, weighed, then dry ashed in a muffle furnace at 550 °C for 2 hours and reweighed. Ashed mass was subtracted from dry mass to obtain the ash-free dry mass (AFDM) of each sea urchin. For each chamber, we multiplied the recorded DO concentrations by seawater volume to obtain total DO and extracted the slope of the linear regression of total DO by time as an estimate of oxygen consumed per second (mg/s) for each measurement. This value was divided by each sea urchin’s AFDM to determine mass-specific oxygen consumption rates (mg/g/s). For each individual sea urchin, we expressed oxygen consumption rates at each experimental timepoint as proportions of the baseline rate. We used 1-sample t-tests to assess if mass-specific oxygen consumption rates at each timepoint differed from the baseline rate.

### 3-day grazing rates

Sea urchin kelp consumption rates represent two different processes: food intake for the individual sea urchins, and potential kelp removal in kelp forest ecosystems. To assess the effects of hypoxia exposure patterns on these functions, we measured grazing rates of *S. purpuratus* over three days. Experiments were conducted in 5.7-L perforated plastic chambers divided in half by 8 × 8 mm plastic mesh to create paired compartments. 64 sea urchins (test diameter 29–34 mm) were each placed in one half of an experimental chamber along with 30 g of fresh, pre-weighed *Macrocystis pyrifera* blades. The other side of each chamber was stocked with the same amount of kelp in order to measure autogenic changes in kelp biomass. We randomly distributed the experimental chambers among eight tanks, which were in turn randomly assigned to the four DO treatments, to give a total of 16 replicate sea urchins per treatment, divided between two tanks.

After three days, we removed sea urchins and kelp from the experimental chambers. We measured the AFDM of each sea urchin in the same way as in the metabolic rate trials. We spun each kelp sample 12 times in a salad spinner^37^ and recorded its wet mass. We subtracted the final kelp mass from the sea urchin chamber from the initial kelp mass, adjusted for the percentage autogenic mass change that was measured in the other half of the chamber (<5% for all replicates), then divided this value by the sea urchin ash-free dry mass to obtain the mass-specific grazing rate (g/g/d). We analyzed mass-specific grazing rates using analysis of variance (ANOVA), with DO Treatment as a fixed factor and Tank as a random factor nested within the DO treatment. Based on Shapiro-Wilk and Bartlett’s tests, the assumptions of normality and homoscedasticity were met (p > 0.17 for all tests).

### 9-week exposure

We assessed grazing, growth, calcification, and reproductive investment in sea urchins over nine weeks of exposure to the four DO treatments from July to September 2017, which corresponds to the season of maximal reproductive investment (gonad production^38^). 96 sea urchins of reproductive size (36–43 mm^39^) were weighed in air and in seawater to obtain initial wet mass and buoyant mass (an estimate of test and spine mass). Individual sea urchins were each randomly assigned to labelled halves of 48 5.7-L perforated experimental chambers, each divided in two halves with plastic mesh, and the chambers were randomly distributed among eight tanks and four DO treatments, such that there were 24 replicate sea urchins in each treatment, divided between two tanks. Sea urchin diameter, wet mass, and buoyant mass did not differ between the treatments prior to the start of the treatment exposures (Nested ANOVA, p > 0.28 for all variables).

Sea urchins were fed *ad libitum* with pre-weighed rations of *M. pyrifera* blades twice a week. At each feeding, the unconsumed portion of the previous ration was removed, spun dry, and weighed to quantify grazing. We also measured percentage autogenic kelp mass change at four time points during the experiment by placing control kelp rations into identical experimental chambers without sea urchins. This value did not differ significantly between treatments (F_3,4_ = 0.19, p = 0.90) or timepoints (F_3,108_ = 0.51, p = 0.68) based on a split-plot ANOVA, so we used the mean autogenic percentage change (1.27%) in our calculations of sea urchin grazing at each time point. We summed the grazed kelp mass across all time points to obtain the cumulative kelp consumption of each sea urchin over the nine weeks. We assessed differences in cumulative kelp consumption using a nested ANOVA, with DO Treatment as a fixed factor and Tank as a random factor nested within the DO treatment. The data were log-transformed to meet the assumptions of normality and homoscedascity (Shapiro-Wilk and Bartlett tests, p < 0.45 for all variables).

We measured sea urchin test diameter, wet mass, and buoyant mass after 35 and 64 days of exposure to the different DO treatments and calculated the percentage growth in each variable. Starting at 35 days, we also conducted a spine regeneration assay^40^ on half the sea urchins. We used dissecting scissors to amputate two complete rows of spines from adjacent ambulacral sections on each sea urchin. Regrowing spines were measured using digital calipers at the end of the experiment, after 29 days of regrowth. Because regeneration was highly uneven among spines and fewer than three spines were fully regrown on any one individual, we measured the length of the three longest regrown spines on each sea urchin and calculated the mean length of these regrown spines for each individual. We assessed differences in the percentage growth of test diameters, wet mass, buoyant mass, and in spine regrowth using nested ANOVAs, with DO Treatment as a fixed factor and Tank as a random factor nested within the DO treatment. All data met the assumptions of normality and homoscedascity (Shapiro-Wilk and Bartlett tests, p > 0.25 for all variables).

At the end of nine weeks, we dissected all individuals, removed coelomic fluid and gut contents, and carefully separated the gonad from the rest of the body. We measured the AFDM of the gonad and the remaining sea urchin body separately and calculated gonad indices by dividing gonad AFDM by the total sea urchin AFDM. We assessed differences in gonad indices using a nested ANOVA with DO Treatment as a fixed factor and Tank as a random factor nested within the DO Treatment.

## Results

### Short-term oxygen consumption rates

Patterns of sea urchin oxygen consumption varied between the four dissolved oxygen (DO) treatments (Fig. 1B). In the ambient treatment, rates of sea urchin oxygen consumption showed some fluctuations, but remained similar to their baseline rates throughout most of the experiment. Under constant exposure to low DO, rates of sea urchin oxygen consumption initially declined by an average of 20.4%, but returned to their baseline rate by the 13.5-hour timepoint, suggesting an ability to compensate for low oxygen availability. The patterns of oxygen consumption in both variable exposure treatments generally reflected temporal fluctuations in DO concentrations. Oxygen consumption was lower than baseline rates during the periods of low DO, and was similar to, or higher than, baseline rates during the periods of ambient DO. In the 6-hour variable exposure treatment, sea urchin oxygen consumption exceeded baseline rates at the timepoints immediately after DO concentrations returned to ambient levels. In contrast, rates of oxygen consumption never exceeded baseline rates during the shorter, 3-hour variable exposures.

### Short- and long-term grazing

**Figure 2:**
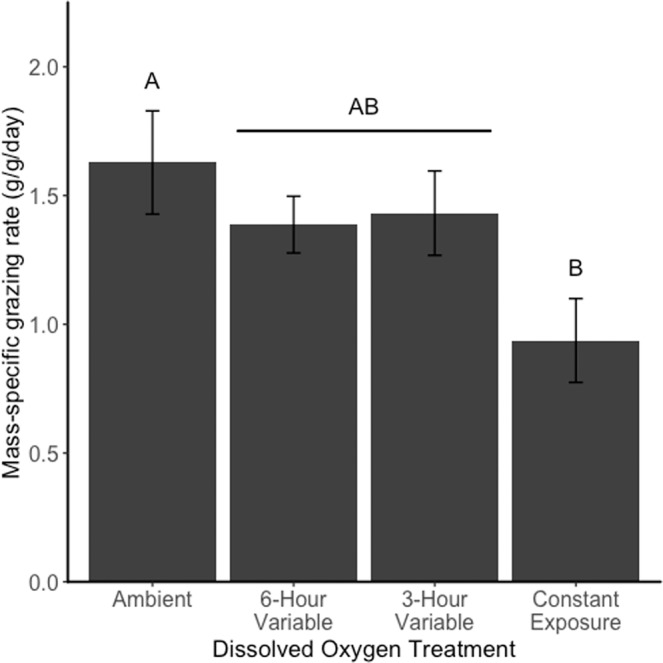
Mass-specific kelp grazing rates (g/g/day) of sea urchins in each dissolved oxygen treatment, measured over a three-day experimental period. Error bars represent the standard error of the mean. Letters indicate differences in mass-specific kelp grazing based on Tukey HSD tests.

**Figure 3:**
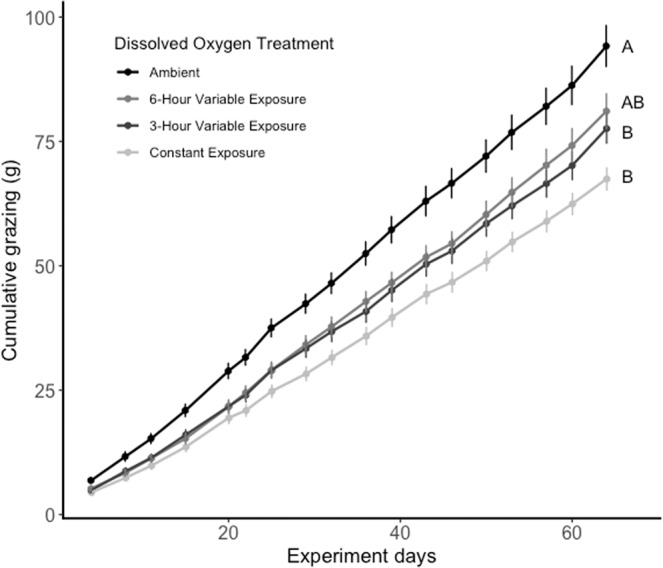
Cumulative kelp consumption (g) of sea urchins in ambient dissolved oxygen (DO) conditions, constant exposure to low DO, 3-hour variable exposure to low DO, and 6-hour variable exposure to low DO over the 64-day experiment. Error bars represent the standard error of the mean. Letters indicate differences in mean final cumulative kelp consumption based on Tukey HSD tests.

Sea urchin mass-specific grazing rates (F_3,59_ = 4.07, p = 0.011) and per-capita grazing rates (F_3,59_ = 4.27, p = 0.0028) varied significantly across DO treatments during the 3-day exposure (Fig. 2). In general, kelp consumption decreased under exposure to low DO. Post-hoc Tukey tests showed that by both measures, sea urchins in the ambient treatment consumed more kelp than those under constant exposure to low oxygen. Sea urchins under both variable exposure regimes consumed an intermediate amount of kelp, not significantly different from either the ambient or constant exposure treatments. Cumulative kelp consumption over 9 weeks also varied across DO treatments (F_3,4_ = 7.47, p = 0.041). Sea urchins in the constant exposure and 3-hour variable exposure treatments consumed significantly less kelp than sea urchins in the ambient treatment, while sea urchins in the 6-hour variable exposure treatment consumed an intermediate amount of kelp (Fig. 3).

### Survival and growth

**Figure 4:**
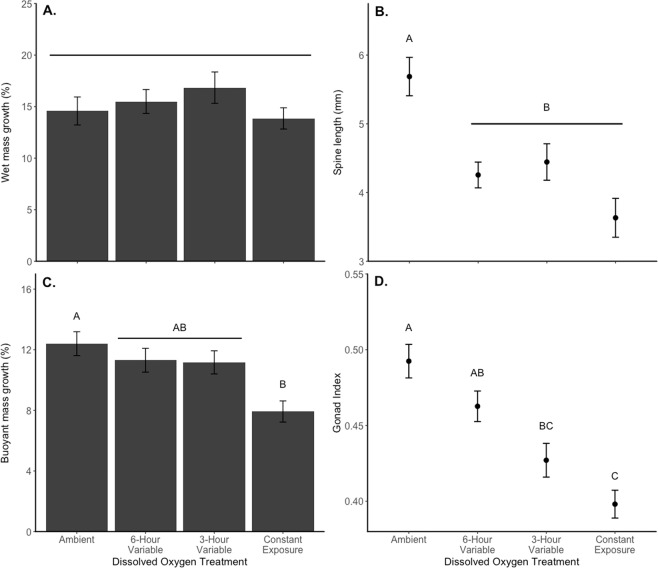
Growth, calcification, and reproductive investment of purple sea urchins in each dissolved oxygen treatment. (**A**) Percentage wet mass growth after 65 days; (**B**) Length of regrown spines after 30 days; (**C**) Percentage buoyant mass growth after 65 days; (**D**) Final gonad index after 65 days. Error bars represent the standard error of the mean and letters indicate differences in mass-specific kelp grazing based on Tukey HSD tests.

No mortality occurred in any of the treatments over 9 weeks, indicating that any hypoxia impacts were sublethal. Total growth was not significantly affected: there were no significant differences in percentage wet mass growth across all DO treatments after 35 days (F_3,4_ = 3.33, p = 0.14) or after 64 days (F_3,4_ = 0.968, p = 0.49, Fig. 4A). On average, sea urchins had an increase of about 15% in wet mass and about 2.5% in test diameter at the end of 9 weeks, with no among-treatment differences in test diameter growth (F_3,4_ = 1.15, p = 0.43).

### Calcification

Spine regrowth, a measure of calcification, was significantly different among DO treatments (F_3,4_ = 11.27, p = 0.02, Fig. 4B). Sea urchins in all three low DO exposure regimes had lower spine regrowth compared to the sea urchins in the ambient treatment (post-hoc Tukey test). Percentage increase in sea urchin buoyant mass also differed among DO treatments. This effect was marginally significant after 35 days (F_3,4_ = 6.09, p = 0.057) and statistically significant after 64 days (F_3,4_ = 7.27, p = 0.043, Fig. 4C). Sea urchins that had spines amputated gained less buoyant mass (F_1,84_ = 19.32, p < 0.001) but there was no interaction between DO treatment and spine amputation (F_1,84_ = 0.16, p = 0.92). Post-hoc Tukey tests indicated that buoyant mass growth for sea urchins in the constant low exposure treatment was significantly lower than for those in the ambient treatment, and sea urchins in both variable treatments regimes had intermediate levels of buoyant mass growth.

### Reproductive investment

Sea urchins’ final gonad indices were significantly different across DO treatments (F_3,4_ = 7.05, p = 0.045, Fig. 4D), with exposures to low DO having negative impacts on reproductive investment. A post-hoc Tukey test showed that sea urchins in the ambient treatment had significantly higher gonad indices compared to those in the constant exposure treatment. Sea urchins in the two variable exposure treatments had mean gonad indices that were between the gonad index values for the ambient and constant exposure treatments. The specific regimes of variable exposure to low DO had different effects on reproductive investment sea urchins in the 6-hour variable exposure treatment had gonad indices more similar to those in the ambient treatment, whereas those in the 3-hour variable exposure had gonad indices more similar to those in the constant treatment.

## Discussion

As climate change alters both the intensity and variability of physiological stressors in ecosystems, it is particularly important to understand the broad impacts of sublethal exposures and the role of realistic patterns of variability in mediating these impacts. Our study shows that sublethal exposures to low DO concentrations have negative impacts on multiple key biological processes in kelp forest sea urchins, and that these impacts are differentially mediated by the temporal pattern of exposure. To our knowledge, this is the first direct investigation of how different patterns of variability can modulate organisms’ responses to a physiological stressor in a naturally variable coastal upwelling system. Critical next steps will be to test the effects of variable exposure to multiple stressors, and subsequently their possible ecosystem-level consequences under field conditions.

Exposure to sublethal levels of hypoxia had negative impacts on purple sea urchins at both short (days) and longer (weeks) time scales. In our 3-day experiments, sea urchins that experienced constant exposure to low DO consumed less kelp compared to those under ambient DO conditions, but generally maintained stable respiration rates after an initial temporary decrease. Because these responses represent reduced food intake without a corresponding decrease in metabolic rates, they suggest that sublethal DO levels lead to a reduced energy budget. Our 9-week experiment suggests that this reduced energy budget has negative impacts on the key processes of calcification and reproduction, with sea urchins in constant low DO conditions showing slower skeletal growth and spine regrowth (calcification), and smaller gonads (reproductive investment), compared to those under ambient conditions. The reduced calcification rates and gonads were not reflected in total wet mass growth rates, likely because most of a sea urchin’s wet mass comes from the its large volume of coelomic fluid, which can obscure patterns of growth in the other body components. Gonad growth can simply displace coelomic fluid with gonad tissue, and have no effect on total wet mass^41,42^. Our results are similar to findings from aquaculture studies that showed reduced food intake and gonad production in green sea urchins exposed to constant low oxygen^43^, and they are also consistent with a broader pattern of reduced reproductive processes in nearshore fish and invertebrates under long-term sublethal hypoxia^7,44–46^. Taken together, our data suggest that critical processes like reproduction may be sensitive to hypoxia even when no effects on survival and growth are apparent. Furthermore, because the longer-term exposures occurred during the season of maximal reproductive investment^38^, trade-offs between reproductive and growth energy investment could result in greater impacts of physiological stress on reproduction than on growth.

The observed organism-level responses can have direct implications for impacts at the population and ecosystem levels. Reduced reproductive capacities of individuals can lead to decreased fertilization and recruitment in sea urchin populations, particularly if they are already at lower densities (*i.e*., Allee effects^47^) Sea urchin spines can aid in protection against predators and/or physical forces^48^, so a reduced ability to regenerate damaged spines may have impacts on mortality rates in the longer term. Kelp consumption by sea urchins is a functionally important process in kelp forest ecosystems^49^, so the modulation of sea urchin grazing by sublethal hypoxia could influence kelp forest dynamics and ecosystem ‘tipping points’^50^. Furthermore, sea urchins’ gonads are the main source of their nutritional value to predators^51^, so sublethal hypoxia impacts on gonad production could also decrease the transfer of energy to higher trophic levels in the kelp forest food web. Therefore, exposures to sublethal hypoxia have potentially wide-ranging effects on sea urchin populations and on kelp forest ecosystem structure and dynamics.

Under variable exposures to sublethal hypoxia, sea urchin responses generally fell in between the responses for those in the ambient and constant exposure treatments. This consistent pattern suggests that the magnitude of impact may be mediated by total (cumulative) exposure duration, although this experimental design did not allow us to fully distinguish the effects of duration and variability between the constant exposure and variable exposure treatments. Importantly, sea urchins did not perform more poorly under variable versus constant exposures to sublethal stress, as has been found for other species^17^. Therefore, constant exposure regimes, which are much more commonly used in experiments, could represent a worst-case scenario of responses for these organisms, even though they are uncommon in the field. However, we also saw that different biological processes were differentially affected by the exposure patterns tested. For example, spine regeneration appeared to be the most sensitive to low DO conditions: sea urchins exposed to all three temporal patterns of sublethal hypoxia exhibited similar decreases in spine regeneration.

Different biological processes also responded differently to different variability patterns. Grazing, growth, and calcification responses were similar among sea urchins in the 3-hour and 6-hour variable exposure treatments, suggesting that these processes may be more sensitive to total exposure duration than to the pattern of exposure. However, sea urchins that spent longer continuous periods of time in ambient oxygen conditions produced larger gonads, suggesting that sea urchin reproductive processes may be sensitive to higher-frequency patterns of variability in sublethal hypoxia. Therefore, present-day spatial differences in the temporal patterns of hypoxia exposure^19,21,22,52^ and future projected changes in DO exposure regimes^27–29^ could produce population-level effects (through reproduction) and ecosystem-level effects (*e.g*., trophic energy transfer) through impacts on just one or two particularly sensitive organism-level responses, such as gonad production. More generally, these results show that different processes may be resilient to hypoxia, or, on the contrary, close to a tipping point of rapid change. These results demonstrate that it is important to consider multiple key biological responses to variable stressor exposure regimes in order to understand climate change impacts on populations and ecosystems and how they may vary spatially.

We could not compare sea urchin metabolic rates from the variable exposure treatments with those from the ambient and constant exposure treatments, or infer patterns in energy budgets because our measured oxygen uptake rates might not be an accurate proxy for sea urchin metabolic rates under variable DO conditions. Sea urchins lack specialized respiratory organs or pigments, and transport of DO to the internal tissues occurs by relatively slow and inefficient diffusion across the tube feet into the ambulacral and coelomic fluids^53^. As a result, the highly variable oxygen uptake rates that we recorded under variable DO conditions could be driven by the changing concentration gradient between the seawater and the ambulacral and coelomic fluids, rather than by metabolic demand^42,53^. Direct measurements of DO concentrations in coelomic fluid^41,42^ will be needed to assess actual sea urchin respiration rates under variable DO conditions. Such measurements may also help to shed light on mechanisms for the observed patterns in other response variables, since most sea urchin tissue is supplied with oxygen from the coelomic fluid.

Climate change impacts on organisms and ecosystems are likely to be multifaceted, involving changes to the both the intensity and variability of multiple physiological stressors^54^. Our study explores, for the first time, the impacts of different patterns of variability in sublethal exposure to an understudied stressor, upwelling-driven hypoxia. We show that these patterns of variability can modulate the negative impacts of hypoxia on ecologically important sea urchins in kelp forests. We find that reproductive investment is the process that appears to be most sensitive to hypoxia, as well as to its exposure regime, with potentially important population and ecosystem level consequences. A remaining challenge is to understand how the changing patterns of variability in multiple environmental stressors will interact to impact organisms and ecosystems, and whether predictions based on laboratory studies hold under field conditions. For example, upwelling-driven hypoxia tends to occur in phase with low temperature and low pH, which can each have direct impacts on sea urchin metabolism, grazing, calcification, and reproduction^55–58^. Experimental studies that integrate variable exposure patterns across such different co-occurring stressors will be needed to understand their potentially interactive effects on species and ecosystems^59,60^, as we move towards a more realistic and societally useful understanding of climate change impacts^54,61^.

## Data Availability

The datasets generated during and/or analysed during the current study are available in the Github repository, https://github.com/lowhnn/variable_exposure.
